# Accounting for polarization in the calibration of a donut beam axial optical tweezers

**DOI:** 10.1371/journal.pone.0193402

**Published:** 2018-02-23

**Authors:** Russell Pollari, Joshua N. Milstein

**Affiliations:** 1 Department of Chemical and Physical Sciences, University of Toronto Mississauga, Mississauga, ON, Canada; 2 Department of Physics, University of Toronto, Toronto, ON, Canada; Universidad Miguel Hernandez de Elche, SPAIN

## Abstract

Advances in light shaping techniques are leading to new tools for optical trapping and micromanipulation. For example, optical tweezers made from Laguerre-Gaussian or donut beams display an increased axial trap strength and can impart angular momentum to rotate a specimen. However, the application of donut beam optical tweezers to precision, biophysical measurements remains limited due to a lack of methods for calibrating such devices sufficiently. For instance, one notable complication, not present when trapping with a Gaussian beam, is that the polarization of the trap light can significantly affect the tweezers’ strength as well as the location of the trap. In this article, we show how to precisely calibrate the axial trap strength as a function of height above the coverslip surface while accounting for focal shifts in the trap position arising from radiation pressure, mismatches in the index of refraction, and polarization induced intensity variations. This provides a foundation for implementing a donut beam optical tweezers capable of applying precise axial forces.

## Introduction

Optical tweezers are a versatile instrument for applying forces to the microscopic world and have emerged as the most precise tool for performing force spectroscopy experiments on biological molecules [1, 2]. Most optical tweezers make use of a tightly focused Gaussian beam; however, advancing methods in focal spot engineering have led to a range of novel optical traps, heralding a new generation of optical tweezers [3]. For instance, donut beams (also known as vortex beams or Laguerre-Gaussian beams) can be used to apply optical torques [4], and improve the axial trapping efficiency by reducing the radiation pressure along the optical axis [5–7]. While these unique optical traps can be employed for micro-manipulation, they have yet to be applied to precision force measurements. Here we focus on developing donut beam optical tweezers into a tool for performing axial force spectroscopy (i.e., along the direction of laser propagation) [8]. The outstanding challenge is the precise calibration of such a device, in particular, calibration of the axial trap strength and the height of the trap at varying displacements above the coverslip.

A Gaussian laser can be readily converted to a “donut-like” beam by introducing a spiral phase plate into the beam path of the form:

$$\begin{array}{c} \psi =-l\phi , \end{array}$$ (1)

where *ϕ* is the azimuthal angle and *l* is an integer representing the topological charge. The result is a helical wave front with a phase singularity at the centre that produces a dark region along the optical axis. A more flexible way to introduce a spiral phase is with a phase-only spatial light modulator (SLM); the advantage being that the SLM can dynamically adjust the imprinted phase.

However, when focusing such a beam through a high-numerical aperture (N.A.) objective, polarization effects can significantly affect the intensity and phase profile [9, 10]. Numerical simulations of the tight focusing of a donut beam through a high-N.A. objective, using vectorial Debye theory, reveal that the presence of the characteristic dark central core depends on the polarization of the beam [11–14]. When the polarization of the beam has the same handedness as the topological charge of the phase, the intensity in the centre of the beam goes to zero. However, for linearly polarized light, the central dark spot begins to fill with light, and when the phase and polarization are anti-aligned, the intensity in the centre fills in significantly.

While these polarization effects are not visible in the far-field, so are difficult to directly image, we demonstrate their influence in altering the axial trapping strength. Jeffries *et al*. [13] previously showed that the axial trap strength of a donut beam tweezers is affected by the choice of right- or left-handed circularly polarized light. However, the axial trap strength is also dependent upon the location of the trap above the coverslip, and changing the polarization of the trap laser will alter the laser pressure, shifting the axial trap position. In this manuscript we explain how to correct for these effects. We show how surface effects can be used to precisely locate the height of the optical trap as the trap is moved axially above the plane of the coverslip, while accounting for the focal shift (in trap position) that arises due to radiation pressure, mismatches in the index of refraction, and polarization induced intensity variations. As expected, trapping with circularly polarized light aligned with the topological charge showed an increase in axial trapping efficiency compared to light of the opposite chirality. Our method of calibrating the precise strength and position of the optical trap lays the foundation for a new approach to axial force spectroscopy [8] via a donut beam optical tweezers.

## Materials and methods

### 0.1 Optical setup

**Fig 1:**
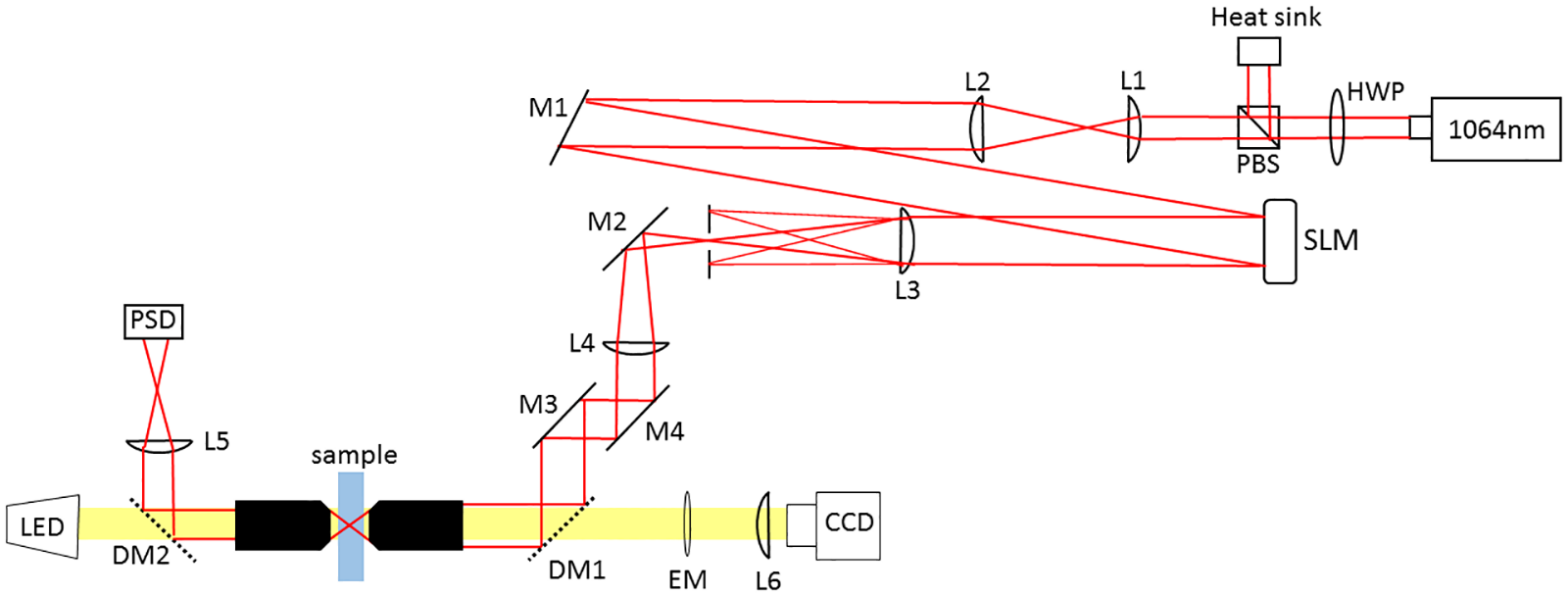
Optical setup. A 1064 nm, near-infrared laser is projected onto a phase-only SLM, and imaged on the back aperture of a high-N.A. objective. An aperture is introduced shortly after the SLM to block all but the first-order diffracted light. The sample plane is illuminated by a white-light LED and imaged both via a CCD and through back focal plane interferometry onto a position sensitive photodiode (PSD).

The optical setup is shown in Fig 1. A 1064nm Nd:YAG laser (4W, Coherent BL-106C) incident on a phase-only spatial light modulator (SLM) (Hammamatsu X10468) is 4-f imaged onto the back focal plane (BFP) of an oil-immersion objective (Olympus PlanApo 100x, 1.4 NA). The laser is vertically polarized using a half wave plate (HWP) and a polarizing beam splitter (PBS), which also allows for manual tuning of the laser power. An iris placed in an intermediate Fourier plane blocks the zeroth order (unmodulated) and any unwanted higher-orders of light reflected off the SLM. The light can be circularly polarized by inserting a quarter wave plate (QWP) before focusing into the sample. A condensor (Olympus LUCPLanFL 40x, 0.75 NA) collects the scattered light and images it onto a position sensitive diode (PSD) (FirstSensor DL100-7-PCBA3) for back focal plane interferometry (BFPI). The axial position of the laser focus *z* is controlled by superimposing a Fresnel lens to the hologram:

$$\begin{array}{c} \phi \left(x^{\prime },y^{\prime }\right)=\frac{2\pi }{\lambda f^{2}}z\left(x^{\prime 2}+y^{\prime 2}\right), \end{array}$$ (2)

where *x*′,*y*′ are the pixel coordinates on the SLM, *λ* is the laser wavelength, and *f* is the focal length of the objective.

To create a donut beam, Eq (1) is superimposed on the SLM with a blazed grating designed to displace the beam a distance *x* from the optical axis, ensuring no interference from unmodulated light reflected off the SLM. The superposition is often referred to as a forked grating:

$$\begin{array}{c} \psi =-l\phi +2\pi \frac{x^{\prime }x}{\lambda f}, \end{array}$$ (3)

where *x*′ is the SLM coordinate, *λ* is the wavelength, and *f* is the focal length of the Fourier transforming lens.

Introducing the phase above generates a field distribution in the Fourier plane that is actually a superposition of radial, higher-order Laguerre-Gaussian modes $LG_{p}^{l}$, which results in multiple rings around a central dark spot (S1 File). There are various approaches to rectifying this issue. For instance, by spatially tuning the efficiency of a blazed phase grating, a phase-only SLM can effectively encode amplitude information [15]. This additional control can be used to modulate all but the lowest order $LG_{0}^{1}$ mode to obtain an essentially pure donut beam; however, we found that purifying the donut beam resulted in a significant reduction in the trap strength (∼50% or more) due to a loss of intensity. For our measurements, we chose to neglect the mode purity corrections to maintain a sufficient trap strength. Since, absent these corrections, the light is already primarily in the $LG_{0}^{1}$ mode, this approximation did not qualitatively affect our results.

### 0.2 Polarization effects

When tightly focusing light with a spatially varying phase profile, such as a donut beam, the polarization of the light can strongly impact its intensity profile at the focus. To better understand the effects of polarization on a donut beam optical trap, the tight focusing of a donut beam though a high-N.A. objective was investigated using vectorial Debye diffraction theory [16]. This approach properly treats the polarization of the trap light, which is not accounted for in scalar diffraction theory (S2 File).

**Fig 2:**
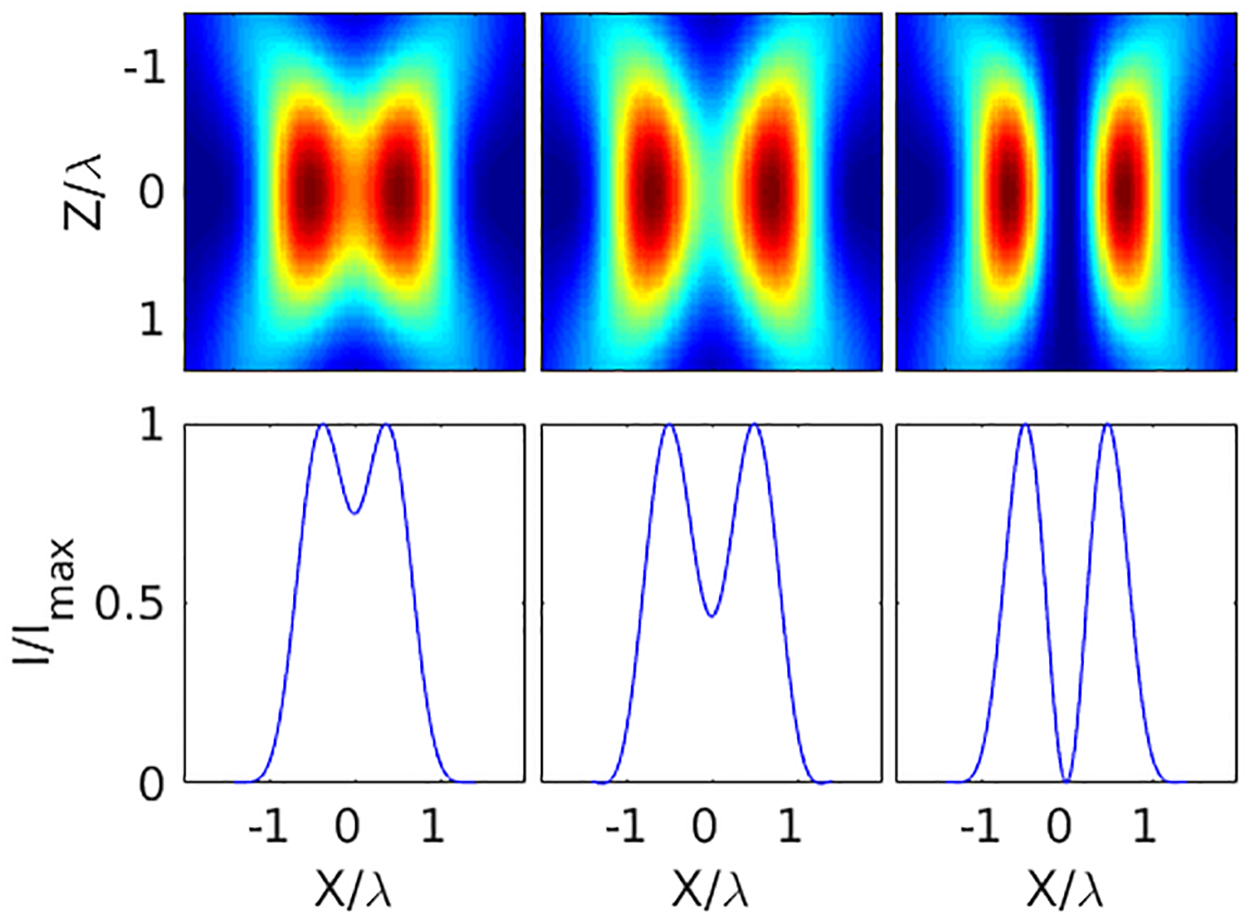
Polarization dependent focal distribution. (Top) Cross-section of the focused light intensity profile along the axial laser direction (N.A. = 1.4). (Bottom) Normalized intensity through the centre of the focal spot. From left to right: LHC (anti-aligned), Linear, and RHC (aligned) polarized incident light.

**Fig 3:**
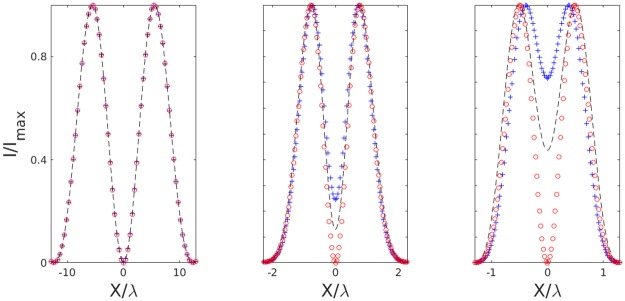
Polarization dependence at varying focal strength. Intensity profiles of $LG_{0}^{1}$ beams with (from left to right) N.A. = 0.1, 1.0, and 1.4. Linear (dashed line), RHC (aligned, circles), and LHC (anti-aligned, crosses). As the N.A. increases, the intensity profile becomes increasingly dependent on the polarization.

Fig 2 shows the axial intensity profile, numerically computed in MATLAB, of the focused laser light for these three polarizations (RHC, LHC, and Linear) as well as intensity cross sections through the centre of each optical trap. When the polarization has the same handedness as the topological charge of an $LG_{0}^{1}$ beam, the central intensity truly goes to zero. Here we have oriented the topological charge so that it is aligned with right-handed circularly (RHC) polarized light. However, when the handedness is of the opposite sign (LHC), the intensity in the centre fills in significantly. And a linearly polarized LG beam has a central intensity somewhere between these two extremes. This same effect is not prominent with lower N.A. objectives, but becomes increasingly important with tighter focusing as is necessary for optical trapping. Fig 3 shows the increasing need to account for polarization effects as the N.A. is increased. In the figure, we present the effects of RHC, LHC, and Linear orientations for increasing N.A. (N.A. = 0.1, 1.0 and 1.4). While at low N.A., the intensity profiles are independent of the polarization, as the N.A. increases, the intensity profiles become strongly dependent on the polarization. We note that these problems could be circumvented by employing more complex polarization schemes, such as azimuthally polarized light, which has been shown to maintain a dark core under tight focusing conditions [17]. The cost, however, is that one needs to generate this novel polarization state.

### 0.3 Relations for optical trap calibration

The standard approach to measuring position within an optical tweezers is through back-focal-plane-interferometry (BFPI) [18]. The Gouy phase shift between light scattered by a trapped micro-bead and unscattered light results in an intensity variation at the back focal plane of the condensor. This variation is proportional to the trapped bead’s axial displacement relative from the trap centre *δz*(*t*), and when imaged on a photodiode is translated into a voltage signal *V*(*t*).

A measure of the detector sensitivity *β* can be used to convert from a voltage to a distance *δz*(*t*) = *βV*(*t*). Both the trap stiffness *κ* and the sensitivity *β* can be determined by measuring the power spectrum of an untethered, trapped bead over the relevant frequencies *f*. The power spectrum takes the form of a Lorentzian as follows [19]:

$$\begin{array}{c} {\left|P\left(f\right)\right|}^{2}=\frac{D_{V}}{2\pi \left(f^{2}+f_{c}^{2}\right)}, \end{array}$$ (4)

with the diffusivity *D*_*V*_ (measured in volts) and the corner frequency *f*_*c*_ extracted by fitting to the data. The trap stiffness and detector sensitivity are determined, respectively, by the relations:

$$\begin{array}{c} \kappa =2\pi f_{c}\gamma , \end{array}$$ (5)

and

$$\begin{array}{c} \beta =\sqrt{\frac{k_{B}T}{\gamma D_{V}}}, \end{array}$$ (6)

where *k_B_T* = 4.1 pN · nm and *γ* is the hydrodynamic drag coefficient. For a microsphere of radius *R*, the axial drag on the particle, close to the coverslip surface, can be approximated by Brenner’s formula:

$$\begin{array}{c} \gamma =\frac{\gamma_{0}}{1-\frac{9R}{8h}+\frac{R^{3}}{2h^{3}}-\frac{57R^{4}}{100h^{4}}+\frac{R^{5}}{5h^{5}}+\frac{7R^{11}}{200h^{11}}-\frac{R^{12}}{25h^{12}}}, \end{array}$$ (7)

where *h* is the trap height, defined as the distance between the trapped bead’s centre and the coverslip, and *γ*_0_ = 6*πηR* is the drag in an infinite medium of viscosity *η* [20]. Eq 7 is clearly nonlinear, so when trapping close to the coverslip surface (roughly, *h* ≤ 3*R*), this calibration becomes acutely sensitive to the height of the trap. We will take advantage of this sensitivity later in calibrating the focal shift of the trap.

## Results

Calibrating a donut beam optical tweezers for use in precision, axial force spectroscopy is challenging. First, due to the radiation pressure, the trap focus is shifted downstream of the laser focus. Second, in large part due to the mismatch in the indices of refraction between the oil/coverslip and aqueous trapping medium, the strength of the optical trap varies as a function of height, which is clearly problematic. We have previously shown that these challenges can be overcome for an axial optical tweezers generated from a Gaussian beam [21]. One can accurately measure the height of the trap from oscillations in the intensity signal due to multiply-reflected light (between a trapped microsphere and the coverslip surface) and even correct for the index mismatch by superimposing an additional hologram.

Unfortunately, a majority of the light that gives rise to the intensity oscillations is lost when working with donut beams, so much so that we are unable to simply apply our previous results of Ref. [21] to the present case. Fortunately, an alternate approach that makes use of surface interactions can be adapted to the current situation. That is, if we can correct for the index mismatch, so that the trap strength remains constant at varying depths above the coverslip, we can apply Brenner’s relation (Eq 7) to accurately calibrate the height of the various donut traps. Note, this approach is often used to estimate the trap height [22], but by adjusting the hologram to maintain a constant trap strength, this approach becomes exact.

In Ref. [21] we showed that correcting for first order spherical aberrations alone was sufficient to correct the index mismatch and achieve a constant axial trap stiffness. Since the correction is independent of the intensity profile, it should also be applicable for optical traps generated by donut beams. At each focal depth *z*, this correction can be imposed by displaying the following phase pattern on the SLM:

$$\begin{array}{c} \Phi_{cor}\left(\rho \right)=Az\left(6\rho^{4}-6\rho^{2}+1\right), \end{array}$$ (8)

where *ρ* is the radial coordinate normalized to the objective’s entrance pupil radius and the constant *A* is empirically determined.

**Fig 4:**
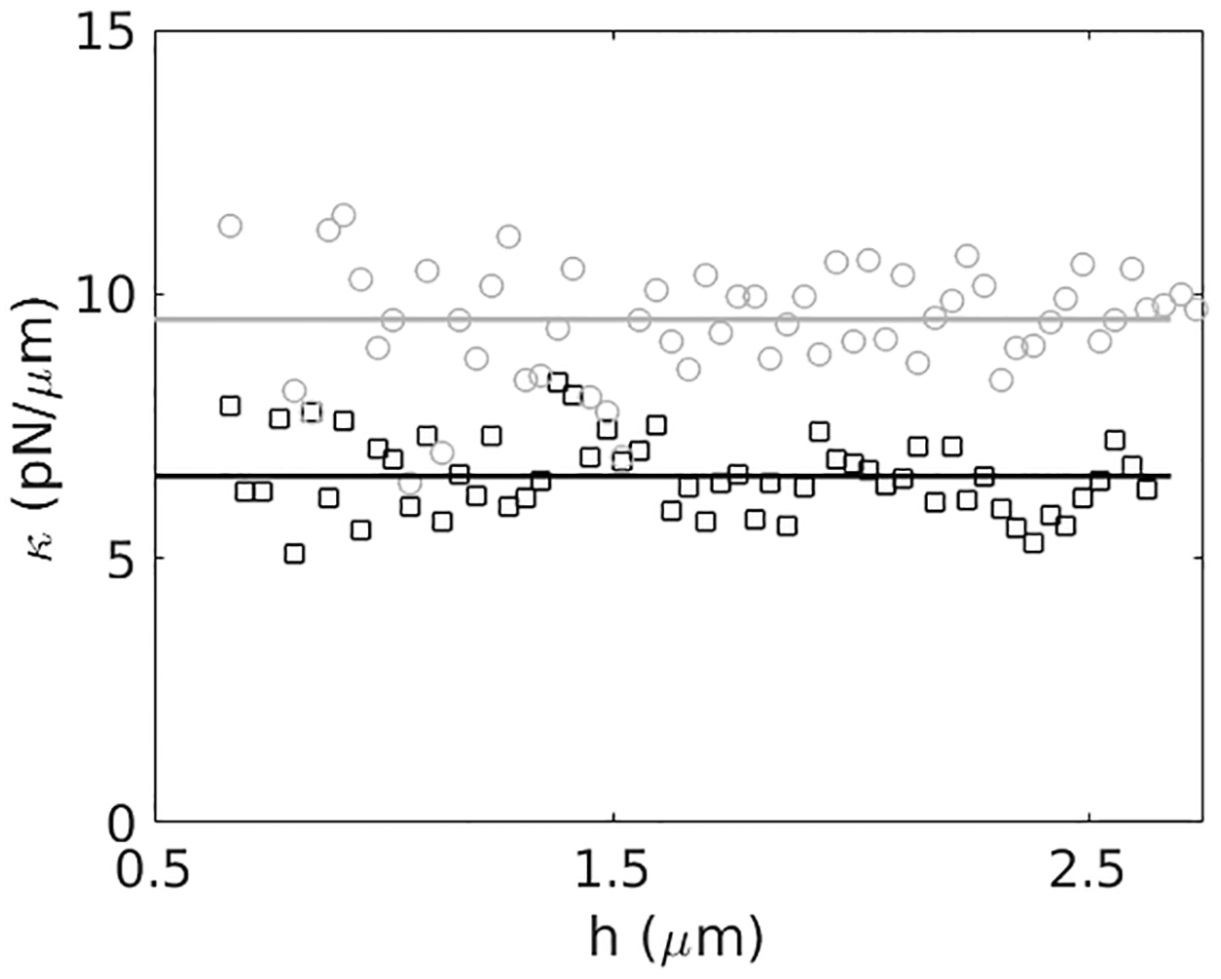
Polarization dependent trap strength. Trap strength *κ* as a function of height *h*, displaying a constant trap stiffness, for LHC (anti-aligned, squares) and RHC (aligned, circles) polarized light. The solid lines are averages of the data at each height. The axial trap strength of the RHC (aligned) polarized trap is 1.44× stronger than the LHC (anti-aligned) polarized trap.

We initially followed this approach to achieve a constant axial trap stiffness for a Gaussian beam (with an *R* = 500 nm polystyrene microsphere), then converted to a circularly polarized donut beam by applying a forked grating to the SLM and inserting a *λ*/4 wave-plate before the objective. Fig 4 shows an experimental measure of the trap strength as a function of axial position for both RHC (aligned) and LHC (anti-aligned) light. With the phase correction of Eq 8 applied, the trap stiffness for both orientations of the donut beam remain constant for at least 3 *μ*m above the coverslip surface. In fact, due to a reduced laser pressure, the axial trapping strength of the RHC polarized trap is ∼44% stronger than that of the LHC trap. Since the trap stiffness *κ* remains constant as a function of the height *h* of the trap, *h* can be directly extracted from a measurement of the corner frequency at each axial position (Eqs 5 and 7), which is how we obtained the horizontal axis in Fig 4. Relative spatial deviations *δz*(*t*) from this height can then be measured through standard BFP interferometry (Sec. 0.3) to precisely track the axial location of a microsphere within a donut beam optical trap.

## Discussion

We have shown that, when generating an optical tweezers with a donut beam, the trapping laser’s polarization must be considered with respect to the imprinted topological charge. The trap strength can be maximized, with a minimum of light intensity at the centre of the donut beam, by employing circularly polarized light aligned with the phase wrap of the topological charge. We have also shown how to use the hydrodynamic drag from the surface to calibrate the precise height of the optical trap above the coverslip. This is achieved by employing Brenner’s formula for the drag as a function of height after tuning the hologram, which generates the donut beam, so that the axial trap strength remains constant over a range of a few microns. With this calibration of trap strength and height, the donut beam tweezers may be used to perform axial force spectroscopy following the same procedure we detail in [21] for a Gaussian beam.

Our results suggest a number of unique implementations of a donut beam optical tweezers that may be realized in the future. For instance, we found that the trap strength increases by almost 50% as the laser polarization goes from being anti-aligned to aligned with the topological charge. This can easily be achieved by simply rotating a quarter-wave plate (as we have done here) or, more rapidly, by employing a liquid crystal retarder. Such an approach would provide a way to apply subtle axial forces without a need to move either the stage or the laser focus, giving rise to a unique, new type of axial optical tweezers.

Another application may be to aid in combining single-molecule fluorescence with optical tweezers. Single-molecule fluorescence can yield direct information on chemical kinetics and local structural changes. Using both techniques in tandem can provide significant new insight into molecular mechanisms inaccessible to either technique alone [23, 24]. Combining the two techniques is not trivial, however. The high intensity light from the trapping laser tends to increase the bleaching rate of fluorescent labels as well as obscure the significantly weaker fluorescence signal [25]. These issues are only significant near the focus of the trapping laser, so can be avoided with sufficient spatial separation of the trap from the fluorescent labels. One may also temporally separate the trapping and excitation beams as the nonlinear processes that drive the enhanced photobleaching are greatly reduced when the fluorophores are not excited [26]. Our work suggests a new approach, similar to the idea of employing donut beams to reduce photodamage proposed in [13], and that is simply to locate a fluorescently labeled molecule within the intensity minimum of the donut beam (with the laser polarization aligned to ensure a true, dark central region). Application of axial forces would maintain the label’s position within the centre of the donut beam. This would ensure that both the trap laser and fluorescence excitation laser are not incident on the labels simultaneously and should mitigate the issues with enhanced photobleaching.

## Supporting information

S1 File Optimization of the mode purity. (PDF)

S2 File Vectorial Debye theory applied to a sharply focused donut beam. (PDF)
