# Supplementary material for: Accounting for polarization in the calibration of a donut beam axial optical tweezers
Source: PLoS One. 2018 Feb 23;13(2):e0193402. doi: 10.1371/journal.pone.0193402 (PMC5825067; doi:10.1371/journal.pone.0193402)
Supplement: S1 File — (PDF) [file pone.0193402.s001.pdf]

S1. OPTIMIZING THE MODE PURITY

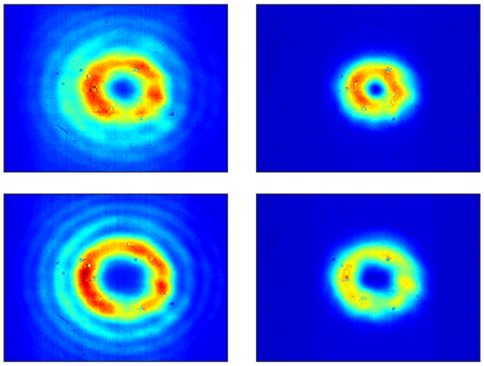

FIG. S1. Far-field intensity profiles (Left) before and (Right) after modifying the hologram to encode amplitude information to purify the LG_0^l mode. The profiles for (Top) $l = 1$ and (Bottom) $l = 2$ are shown.

A Gaussian beam incident on a forked grating with topological charge l produces a field U_l in the Fourier plane that is actually a superposition of Laguerre-Gaussian modes with azimuthal order l and radial orders p:

$$U_l(r,\theta) = \sum_p C_p^l LG_p^l(r,\theta), \tag{1}$$

where C_p^l is a coefficient determining the amplitude of each mode and

$$LG_p^l(r,\phi) \propto (\frac{\sqrt{2}r}{w_0})^{|l|} \exp(-\frac{r^2}{w_0^2}) L_p^{|l|}(\frac{2r^2}{w_0^2}) \exp(-il\phi), \tag{2}$$

where w_0 is the beam waist and L_p^l are generalized Laguerre polynomials [1]. For low values of l, the majority of the power ends up in the $p = 0$ mode, which corresponds to a single ring, but a portion of the intensity goes into higher radial modes [2, 3]. One approach to purifying the lowest mode is to encode amplitude information in the phase-only hologram. This can be achieved by spatially modulating the diffraction efficiency of the blazed grating imprinted on the SLM. There are a number of ways to implement this approach [3, 4]. One of the most efficient ways to encode the amplitude profile A, so as to maintain the intensity of the final hologram, is as follows:

$$\Psi = \psi + f(A)\sin(\psi), \tag{3}$$

where $J_0[f(A)] = A$ and J_0 is the zeroth order Bessel function of the first kind [4]. Thus, single ring intensity profiles are produced by setting $A = |LG_0^l|$. Figure S1 shows the intensity profiles for $l = 1$ and $l = 2$, before and after the addition of the amplitude mask. The higher order radial modes, seen as additional outer rings, vanish with the mask applied.

---

[1] Karimi E, Zito G, Piccirillo B, Marrucci L, Santamato E. Hypergeometric-gaussian modes. Opt Lett. 2007;32(21):30533055.

[2] Sephton B, Dudley A, Forbes A. Revealing the radial modes in vortex beams. Appl Optics. 2016;55(28):78307835.

[3] Ando T, Ohtake Y, Matsumoto N, Inoue T, Fukuchi N. Mode purities of Laguerre-Gaussian beams generated via complex-amplitude modulation using phase-only spatial light modulators. Opt Lett. 2009;34(1):3436.

[4] Arrizon V, Ruiz U, Carrada R, Gonzalez LA. Pixelated phase computer holograms for the accurate encoding of scalar complex fields. J Opt Soc Am A. 2007;24(11):3500 3507.
