# Supplementary material for: Accounting for polarization in the calibration of a donut beam axial optical tweezers
Source: PLoS One. 2018 Feb 23;13(2):e0193402. doi: 10.1371/journal.pone.0193402 (PMC5825067; doi:10.1371/journal.pone.0193402)
Supplement: S2 File — (PDF) [file pone.0193402.s002.pdf]

S2. VECTORIAL DEBYE THEORY

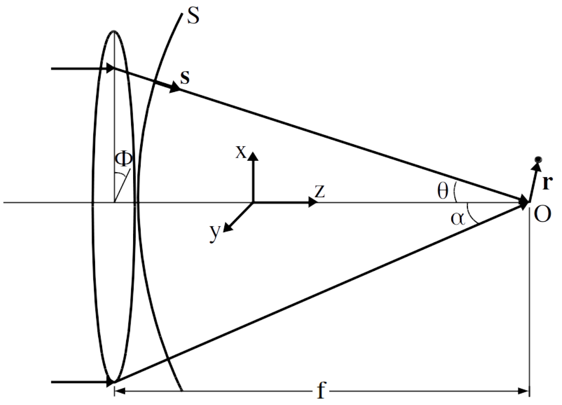

FIG. S2. Geometry for numerical Debye simulations.

The electric field vector near the focus is given by:

$$\mathbf{E}(r,\psi,z) = -\frac{if}{\lambda}\iint \mathbf{E_0}(\theta,\phi)\exp(il\phi)\exp(ikr\sin\theta\cos(\phi-\psi))\exp(ikz\cos\theta)\sin\theta d\theta d\phi, \quad (1)$$

where ϕ is the azimuthal angle, and θ is the angle of the numerical aperture (see Fig S2). $\mathbf{E_0}(\theta,\phi)$ is the electric field vector along a spherical surface S centred on the focal spot (see Fig S2). For circularly polarized light focused through an objective obeying the sine condition [1]:

$$\mathbf{E_0}(\theta,\phi) = A(\theta)\sqrt{\cos\theta}\left[\begin{pmatrix}\cos^2\phi\cos\theta+\sin^2\phi\\ \cos\phi\sin\phi(\cos\theta-1)\\ \cos\phi\sin\theta\end{pmatrix} \pm i\begin{pmatrix}\cos\phi\sin\phi(\cos\theta-1)\\ \sin^2\phi\cos\theta+\cos^2\phi\\ \sin\phi\sin\theta\end{pmatrix}\right], \quad (2)$$

where $\pm$ denotes right-handed or left-handed circular polarization and $A(\theta)$ is the amplitude distribution along the surface S. Inserting Eq. 2 into Eq. 1 yields the electrical field component in all three dimensions:

$$E_x(r,\psi,z) = -\frac{i^m kf}{2}\int_0^\alpha d\theta A(\theta)\sin\theta\sqrt{\cos\theta}\exp(ikz\cos\theta)\times[(1+\cos\theta)J_m(kr\sin\theta)\exp(im\psi) + (1-\cos\theta)J_{m\pm2}(kr\sin\theta)\exp(i(m\pm2)\psi)], \quad (3)$$

$$E_y(r,\psi,z) = -\frac{i^m kf}{2}\int_0^\alpha d\theta A(\theta)\sin\theta\sqrt{\cos\theta}\exp(ikz\cos\theta)\times i[\pm(1+\cos\theta)J_m(kr\sin\theta)\exp(im\psi) \mp (1-\cos\theta)J_{m\pm2}(kr\sin\theta)\exp(i(m\pm2)\psi)], \quad (4)$$

and

$$E_z(r,\psi,z) = -i^m kf \int_0^\alpha d\theta A(\theta)\sin\theta\sqrt{\cos\theta}\exp\left(ikz\cos\theta\right)\times i^{\pm 1}\left[J_{m\pm 1}(kr\sin\theta)\exp(i(m\pm 1)\psi)\right], \tag{5}$$

where $\alpha = \sin^{-1}(N.A./n)$ is the maximal angle of the objective imaged through a medium of refractive index n, and J_m are Bessel functions of the first kind.

---

[1] Gu M. Advanced optical imaging theory. vol. v. 75. Berlin: Springer; 2000.
